# Decision tree based ensemble machine learning model for the prediction of Zika virus T-cell epitopes as potential vaccine candidates

**DOI:** 10.1038/s41598-022-11731-6

**Published:** 2022-05-12

**Authors:** Syed Nisar Hussain Bukhari, Julian Webber, Abolfazl Mehbodniya

**Affiliations:** 1 National Institute of Electronics and Information Technology (NIELIT), Ministry of Electronics and Information Technology (MeitY), Govt. of India, Srinagar, J&K 191132 India; 2 Department of Electronics and Communication Engineering, Kuwait College of Science and Technology (KCST), Doha Area, Kuwait

**Keywords:** Computational biology and bioinformatics, Diseases, Engineering

## Abstract

Zika fever is an infectious disease caused by the Zika virus (ZIKV). The disease is claiming millions of lives worldwide, primarily in developing countries. In addition to vector control strategies, the most effective way to prevent the spread of ZIKV infection is vaccination. There is no clinically approved vaccine to combat ZIKV infection and curb its pandemic. An epitope-based peptide vaccine (EBPV) is seen as a powerful alternative to conventional vaccinations because of its low production cost and short production time. Nonetheless, EBPVs have gotten less attention, despite the fact that they have a significant untapped potential for enhancing vaccine safety, immunogenicity, and cross-reactivity. Such a vaccine technology is based on target pathogen’s selected antigenic peptides called T-cell epitopes (TCE), which are synthesized chemically based on their amino acid sequences. The identification of TCEs using wet-lab experimental approach is challenging, expensive, and time-consuming. Therefore in this study, we present computational model for the prediction of ZIKV TCEs. The model proposed is an ensemble of decision trees that utilizes the physicochemical properties of amino acids. In this way a large amount of time and efforts would be saved for quick vaccine development. The peptide sequences dataset for model training was retrieved from Virus Pathogen Database and Analysis Resource (ViPR) database. The sequences dataset consist of experimentally verified T-cell epitopes (TCEs) and non-TCEs. The model demonstrated promising results when evaluated on test dataset. The evaluation metrics namely, accuracy, AUC, sensitivity, specificity, Gini and Mathew’s correlation coefficient (MCC) recorded values of 0.9789, 0.984, 0.981, 0.987, 0.974 and 0.948 respectively. The consistency and reliability of the model was assessed by carrying out the five (05)-fold cross-validation technique, and the mean accuracy of 0.97864 was reported. Finally, model was compared with standard machine learning (ML) algorithms and the proposed model outperformed all of them. The proposed model will aid in predicting novel and immunodominant TCEs of ZIKV. The predicted TCEs may have a high possibility of acting as prospective vaccine targets subjected to in-vivo and in-vitro scientific assessments, thereby saving lives worldwide, preventing future epidemic-scale outbreaks, and lowering the possibility of mutation escape.

## Introduction

ZIKV is an enveloped virus and is a member of the family Flaviviridae, genus Flavivirus. Its Infection is transmitted through the bite of an infected Aedes mosquito^1^. In 2016, approximately 216,207 cases were reported in Brazil, which is considered an epidemic hotspot, and 8604 babies were born with malformations^2^. So far, 86 nations and territories have reported shreds of evidence of ZIKV infection^2^. The ZIKV infection is spreading rapidly in India^3^ and cases are being reported daily from different states like Kerala, Uttar Pradesh (UP) etc. On 08-11-2021, from just Kanpur city of UP, a total of 89 cases were reported in one day^4^. The vast majority of ZIKV infected persons are asymptomatic. In general, symptoms include moderate fever, joint and muscular soreness, conjunctivitis and headache that lasts 2 to 7 days^5^. The estimated incubation period is 3 to 14 days^5^. ZIKV infection can pass on to pregnant woman's fetus and is the major cause of microcephaly and other congenital abnormalities in growing fetus and newborns^6^.

It is a positive-sense, single-stranded, unsegmented RNA virus. The length of ZIKV genome is 10.7 kilobases. The entire ZIKV genome i.e., a single large protein encodes three (03) structural proteins: envelope (E) protein, a membrane (M) protein and a capsid (C) protein^7^ as well as seven non-structural proteins namely, NS1, NS2A, NS2B, NS3, NS4A, NS4B, and NS5^8^. The envelope (E) glycoprotein is the primary antigenic determinant that facilitates viral entrance by mediating fusion and binding^9^. As a result, the envelope-E glycoprotein has emerged as a prominent target for the development of antiviral therapies and vaccine candidates. Even though there is no vaccine to combat the infection; however, it is recommended to take necessary measures like resting, drinking enough water to remain hydrated, taking paracetamol and acetaminophen to avoid this illness^10^. The primary contributions of the current study are:

- Proposed an ML ensemble model for predicting TCEs of ZIKV as potential vaccine targets.
- As epitope prediction is considered a critical task, the main focus of the study is on accuracy. Other essential parameters which have been taken into consideration are, AUC, sensitivity, specificity, Gini and MCC.
- Compared the proposed model with standard existing ML classifiers, namely decision tree (DT), support vector machine (SVM), neural networks (NN), random forest (RF), and AdaBoost (Ada), where the proposed model outperformed them.
- The consistency and reliability of the proposed ensemble model has been assessed using k-Fold cross-validation (KFCV) method.

The motivations to conduct this study are:

- Using conventional vaccine based on full organism have several drawbacks if immunologically redundant biological components are present. So EBPV is considered safe because only those TCEs are selected for developing EBPV, which are antigenic instead of the whole organism.
- In comparison to in-vivo approaches, the ML-based in-silico approach for TCE prediction of ZIKV would save time for quickly developing the vaccines.
- Existing method for TCE predictions namely, NetMHC^11^ only estimates peptide‘s binding capacity while as a method namely, CTLpred^12^ predicts peptides up to length 9-mers only. So there is a need for an accurate and reliable method that can directly predict if a peptide sequence is a TCE or not. The method so developed should also be able to predict peptides of length greater than 9-mers.
- Also, existing prediction methods are based on SVM and ANN only (single classifier based). However, in the present study, we have developed an ensemble ML model intending to improve the prediction performance, make better forecasts and deliver better results over any single classifier^13^.
- In addition, ZIKV continues to profoundly impact lives across the globe, especially in third-world countries, due to the lack of a vaccine for its treatment and prevention. So keeping this in mind and its recent outbreak in India, vaccine development for this disease is considered a hot research domain for scientists.

As per the literature study, for the prediction of TCEs of ZIKV, various bioinformatics and machine learning based methods primarily, NetMHC^11^ and CTLpred^12^ are currently in use^14^. The NetMHC method built using neural network and SVM classifiers only provides peptide’s binding capacity instead of predicting deterministically whether a peptide in an epitope or not. The method CTLpred predicts in a deterministic manner using NN, SVM and quantitative matrix approaches. However, it can only predict peptides up to 9-mers in length. Apart from NetMHC and CTLpred prediction servers, other in-silico based studies have been proposed to predict TCE of ZIKV. In their research, Yadav et al.^15^ have used a ProPred^16^ immunoinformatics tool to predict MHC class II promiscuous epitopes. It was found that the “YRIMLSVHG” epitope sequence belonging to glycoprotein bound to MHC class II allele “DRB1*01:01” has demonstrated a good binding score. Pandey et al.^17^, have used ZIKV structural and non-structural proteins in their investigation to develop a multi-epitope subunit vaccine utilizing combinatorial immunoinformatics. The subunit vaccine are composed of helper T lymphocytes (HTL) and cytotoxic T lymphocytes (CTL) epitopes. In addition to HTL and CTL epitopes, adjuvants and linkers are also added. In their study, Shahid et al.^18^, employed an immunoinformatics and molecular docking approaches to create a multi epitope-based peptide (MEBP) vaccine. Following prediction, 14 CTL and 11 HTL epitopes were selected which were linked to peptides via AAY and GPGPG linkers, respectively. Prasasty et al.^19^, employed immunoinformatics to identify T-cell epitope candidates in a range of ZIKV proteomes. Specific HLA alleles have been used to map putative TCEs. Using molecular docking, it has been demonstrated that there is a peptide-HLA interaction MHC-II epitopes.

## Methods

### Data

The ZIKV peptide sequences were retrieved from “Virus Pathogen Database and Analysis Resource (ViPR)” maintained by “National Institute of Allergy and Infectious Diseases (NIAID) through the web site: http://www.viprbrc.org/”^20^ in comma-separated values (CSV) file format. The sequences consist of both TCEs and non-TCEs. Only linear peptide sequences were taken into consideration for this study. A total of 12,262 peptide sequences were retrieved, of which 6120 are epitopes and 6142 are non-epitopes. The current problem being binary classification problem; we added a dependent variable called “Class” in both the CSV files. For TCE sequence, the “Class” variable has a value of 1 and for non-TCE a value 0.

### Proposed methodology

**Figure 1:**
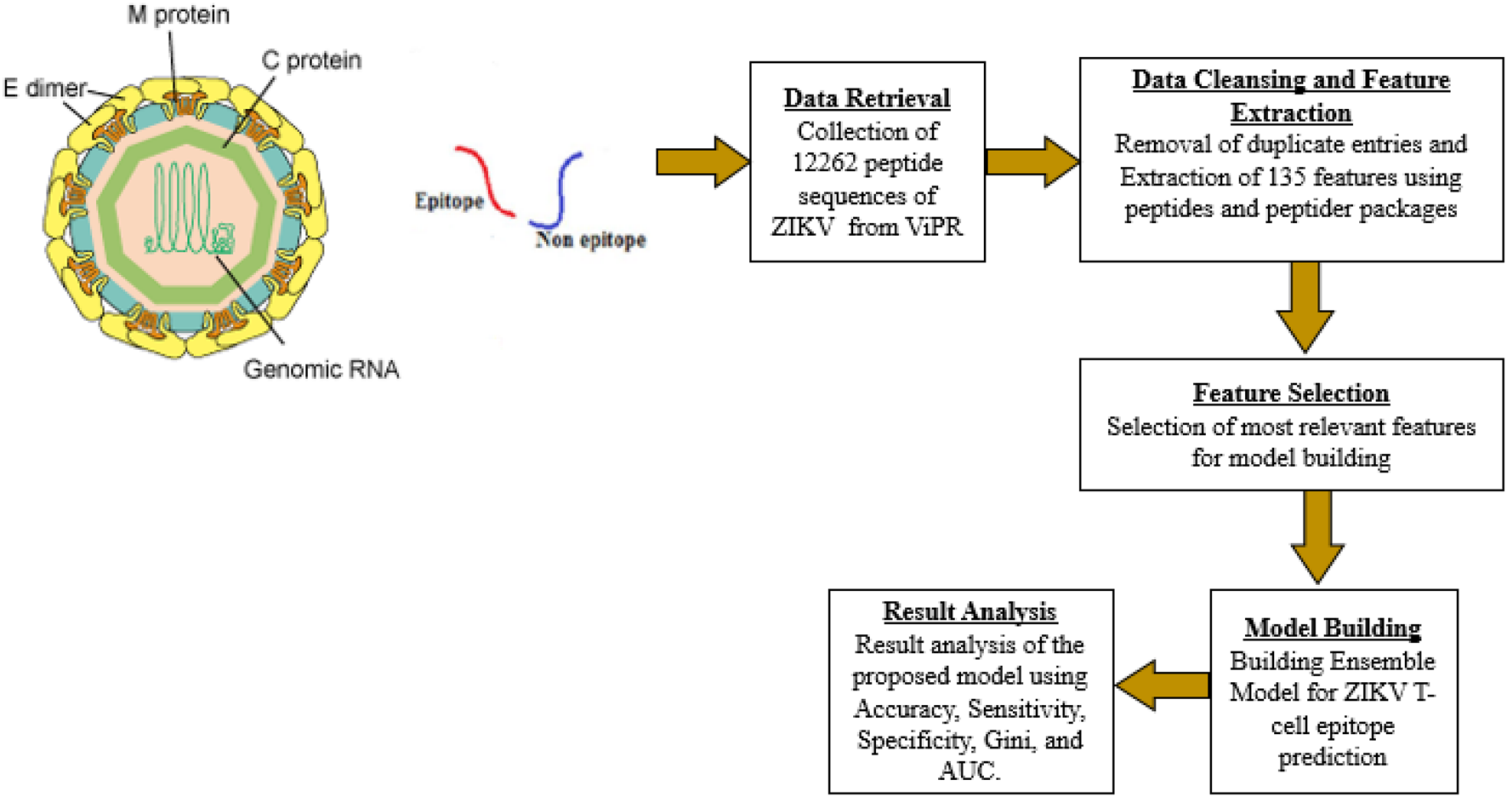
Proposed methodology.

Figure 1 depicts the proposed methodology and is described through the following subsections. The ZIKV particle image was collected from an open access webpage:” https://www.creative-diagnostics.com/Zika-Virus.htm”.

#### Data cleaning and extraction of features

After retrieving TCEs and non-epitope sequences from ViPR, the next step is to extract features. Before performing feature extraction, a few duplicate entries were removed. The physicochemical properties of amino acid sequences act as features in this study. For feature extraction, the R programming packages “peptides”^21^ and the “peptider”^22^ have been used^23^. The collected features from two CSV files were eventually combined into one CSV file. The amino acid physicochemical properties employed in this study are depicted in Table 1. In Table 2, a preview of the dataset is depicted.

**Table 1:** Amino acid physicochemical properties used.

Physicochemical property	Count	Notation
Aliphatic-index	1	F1
Boman-index	1	F2
Insta-index	1	F3
Probability of detection	1	F4
Cross-covariance-index	1	F5
Hmoment-index	2	F6_1, F6_2
Molecular weight	2	F7_1, F7_2
Peptide charge for 45 scales	45	F8_1 to F8_45
Hydrophobicity at 44 scales	44	F9_1 to F9_44
Isoelectric point for 9 pKscale	9	F10_1 to F10_9
Kidera factors	10	F11_1 to F11_10
aaComp	18	F12_1 to F12_18

**Table 2:** Snapshot of the dataset.

Peptide sequence	F1	F2	F3	.	F12_16	F12_17	F12_18	Class
AARVTAIL	29.82	0.86	20.31	.	− 0.76	− 0.38	0.19	1
ADLMGYIPL	12.54	0.34	76.33	.	− 0.12	− 0.10	− 0.05	1
ELAAKLVAL	98.34	3.65	− 8.21	.	− 0.98	− 0.35	− 0.12	1
AARALAHGV	87.12	− 8.36	2.73	.	− 0.64	− 0.73	− 0.76	0
FSIFLLALL	76.0	− 4.54	7.21		− 0.45	− 0.30	− 0.32	0

#### Selection of important features

**Figure 2:**
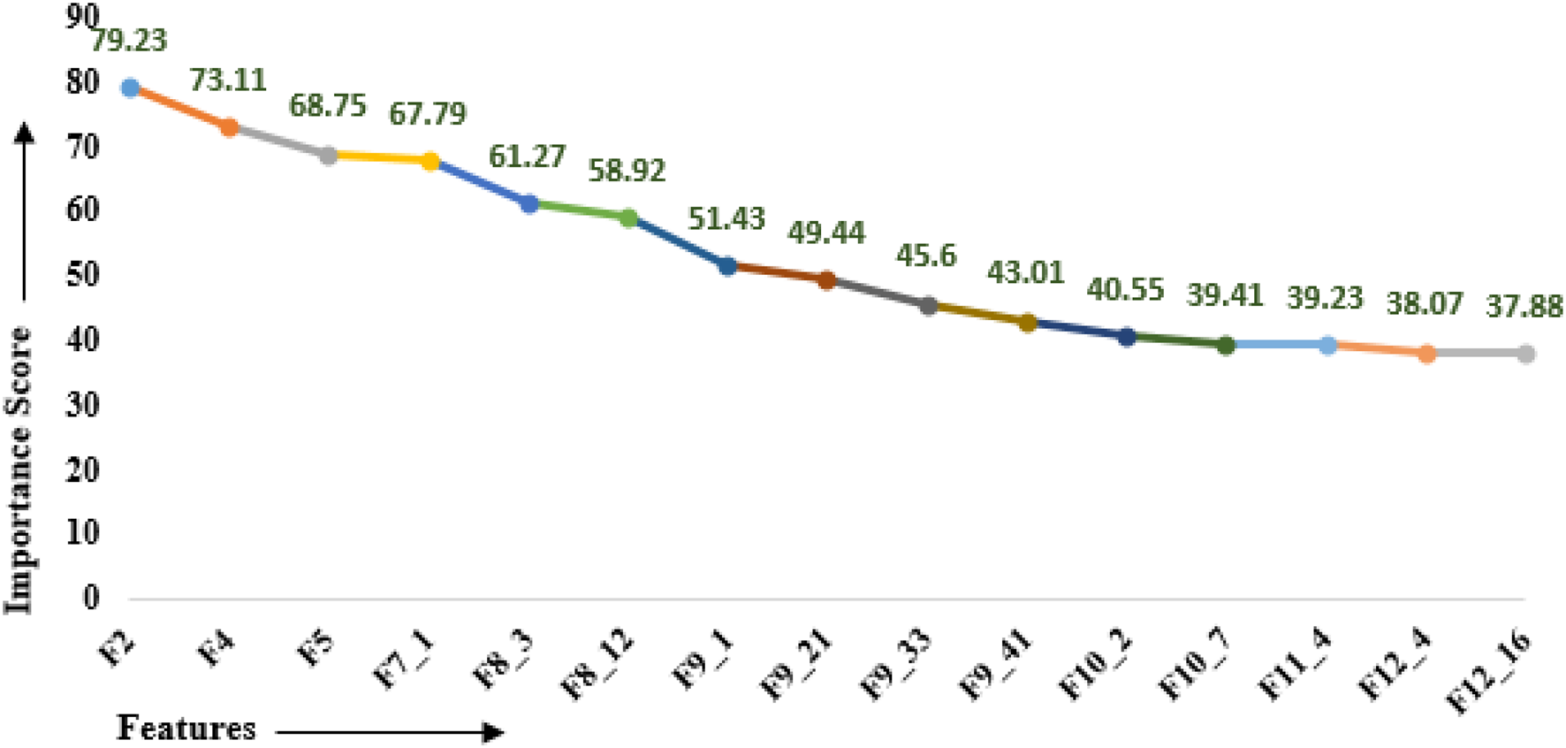
Feature importance line plot.

Feature selection (FS) decreases the number of independent variables when building an ML classifier and is always desirable for two main reasons: reducing computational cost and improving model performance. In this paper, the Boruta^24^ algorithm in R was used for carrying out the FS process. It is a wrapper algorithm that considers the values of minImp, maxImp, medianImp, normHits and meanImp parameters to find the essential features. The Boruta algorithm takes dependent variable i.e., “Class and the dataset consisting of 135 features as input. After applying Boruta, the fifteen (15) top relevant features were returned as shown in Table 3. The features are listed in decreasing order of importance score. Figure 2 depicts the line plot of essential features selected by Boruta. The 15 features are finally used for training and building the proposed ensemble model.

**Table 3:** Important features selected by Boruta.

Feature	Importance score
F2	79.23
F4	73.11
F5	68.75
F7_1	67.79
F8_3	61.27
F8_12	58.92
F9_1	51.43
F9_21	49.44
F9_33	45.6
F9_41	43.01
F10_2	40.55
F10_7	39.41
F11_4	39.23
F12_4	38.07
F12_16	37.88

#### Building voting ensemble model

**Figure 3:**
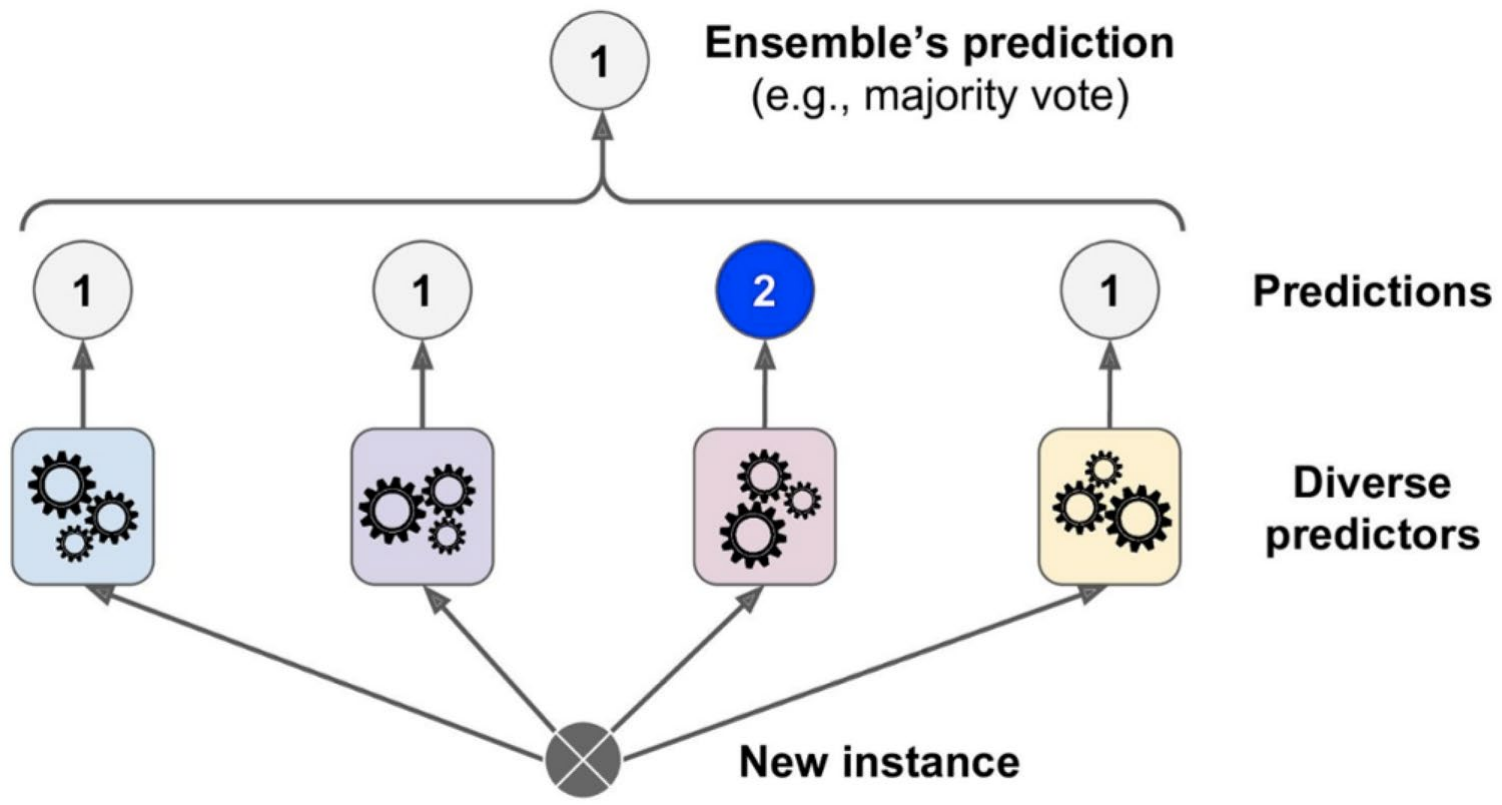
Hard voting classifier prediction.

Ensemble learning (EL) is a technique that combines several classifiers to solve a particular computational intelligence problem. Multiple classifiers, also known as base learners (BLs), are trained, and their predictions are combined as a single output. The main aim of using EL is to improve the classification accuracy of the model^25–27^. An ensemble can be created by training similar BLs using different subsets of the entire training dataset (approach one) or heterogeneous BLs using the same training dataset (approach two). This current study is based on approach one, in which the training dataset has been divided into multiple different splits^28^. A voting ensemble (VE), also called the majority voting ensemble technique, has been used in this study. The VE is an EL model in which predictions from multiple BLs are combined. Voting ensembles are of two types: hard voting and soft voting. In hard voting, the sum of votes from different BLs for class is performed^29^. Then the class having maximum votes is decided as the final class prediction. Forecasted probabilities for class labels from different BLs are added in soft voting, and the class with the highest sum probability is predicted as the final class. Because it outperforms other classifiers, the decision tree (DT) was utilized as the basis for developing a hard voting ensemble model in the current study. Also, DT can deal with high-dimensional data, have high precision, and uses an inductive strategy to learn about characterization^30^. The hard voting ensemble technique used in the current study is shown in Fig. 3.

**Figure 4:**
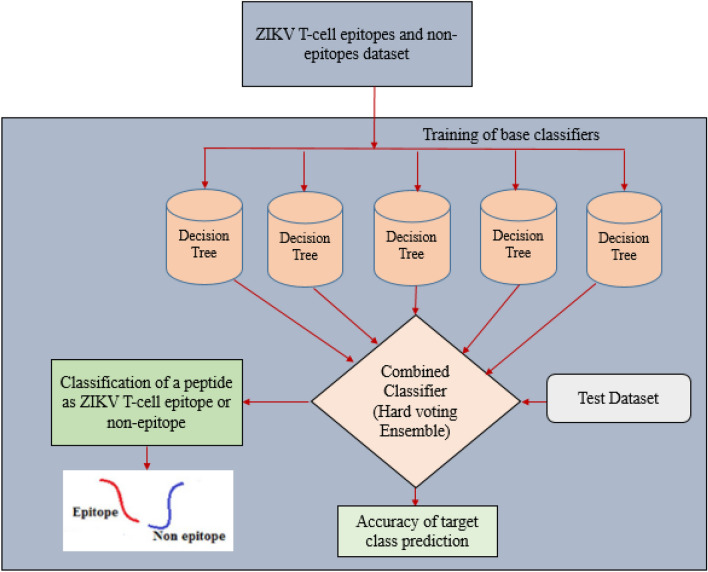
Proposed hard voting ensemble model for ZIKV T-cell epitope prediction.

The proposed hard voting ensemble model based on DT classifier is depicted in Fig. 4. As depicted in Fig. 4, all DT base classifiers have been trained on 80% of dataset and then a hard voting EL technique has been used to combine them. A test data set comprising of 20% data instances randomly picked from each frame have been utilized to evaluate the performance of the proposed ensemble model.

#### Decision tree as base classifier

**Figure 5:**
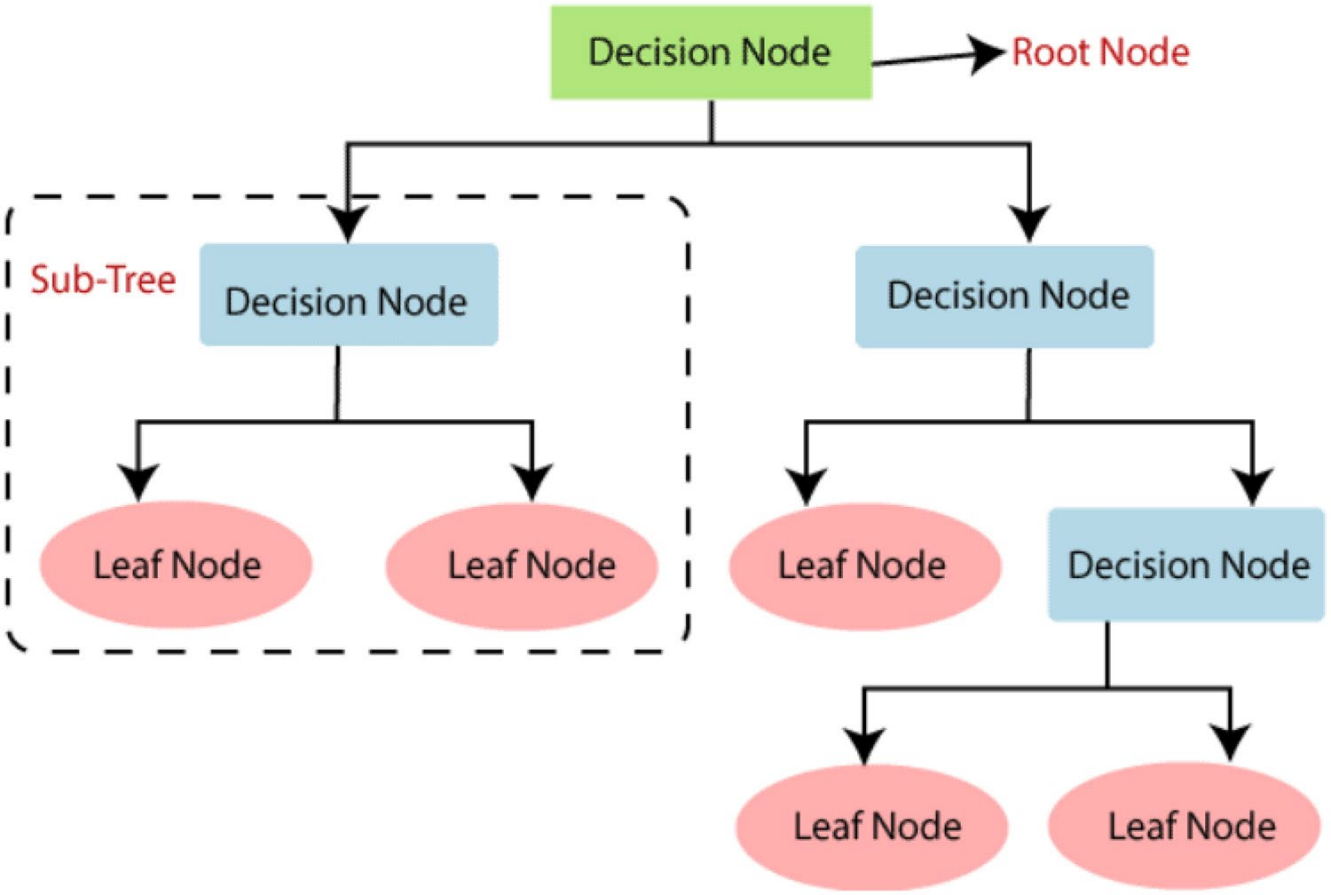
Decision tree classifier.

DT is a supervised learning algorithm used for classification and Regression problems but is principally more popular for classification tasks^31^, as shown in Fig. 5. The reason DT has been used as base classifiers is that its performance for binary classification problems is superior to that of other classifiers. It has a tree-like structure with two types of nodes; an internal node or decision node for making decisions and leaf node representing the output^31^. The branches of the tree represent the selection rules.

In order to infer DT classifier, the rpart() function in R has been used^32^. For performance improvement, tuning of “usesurrogate” and “maxsurrogate” parameters of the DT classifier was performed. The "maxsurrogate" parameter denotes the no. of surrogate splits, while the "usesurrogate" parameter specifies the use of surrogates during the process of split. When both the parameter are set to 0, the computational time is greatly decreased because the surrogate split search occupies nearly half of the processing time. Table 4 lists the classifiers which have been employed for carrying out the comparative analysis with the proposed model along with their parameters. The model was implemented in the R language environment under the “GNU-GPL license”^23^. The prototype for the DT classifier used in this study is: “rpart (formula, train Dataset, maxsurrogate=0, usesurrogate=0)”. Equation (1) shows the model training formula, which outputs a dependent variable “Class” as label, and its input is the corresponding 20 features.

1 $$\begin{gathered} {\text{Class}} \sim {\text{f }}({\text{F2}},{\text{ F4}},{\text{ F5}},{\text{ F7}}\_{1},{\text{ F8}}\_{3},{\text{ F8}}\_{12},{\text{ F9}}\_{1},{\text{ F9}}\_{21}, \, \hfill \\ {\text{F9}}\_{33},{\text{F9}}\_{41},{\text{ F1}}0\_{2},{\text{ F1}}0\_{7},{\text{ F1}}\_{4},{\text{ F12}}\_{4},{\text{ F12}}\_{16}) \hfill \\ \end{gathered}$$

**Table 4:** Illustration of ML classifiers used for comparison.

Classifier	Tuned parameters	R Package
Decision trees^33^	maxsurrogate = 0, usesurrogate = 0	rpart
Neural network^34^	Size = 10, maxit = 100	nnet
Support vector machine^35^	kernel = "rbfdot", type = C-svc”	ksvm
adaBoost^36^	iter = 50, type = ”discrete”, nu = 0.5	ada
Random forest^32^	ntree = 500, mtry = 2	randomForest

#### Predictions by the proposed ensemble model

Classification accuracy of the proposed model was evaluated using test dataset. For building an ensemble, we have used an odd number of DT classifiers to avoid ties. As a result, the evaluation is based on the votes of five DT classifiers, and the class label is predicted using a majority vote approach. The proposed ensemble approach is now capable of predicting any ZIKV peptide sequence. The proposed model correctly predicted all the testing tuples when tested using the test dataset and the results as described in “Results” are accurate and reliable.

## Model evaluation

The process of evaluating a model using various parameters is known as model evaluation. Because predicting TCEs is a task that belongs to binary classification, there are four (04) probable outputs: namely, True Positive (TP), True Negative (TN), False Positive (FP) and False Negative (FN)^37^.

In this study, for evaluating the model, metrics namely accuracy, MCC and Area under receiver operator characteristic curve (AUROC), specificity, sensitivity and Gini have been used^38^. All these metrics are defined in terms of above mentioned four possible outcomes. To examine the consistency and robustness of the proposed model, a validation technique called K-fold cross-validation (KFCV) have been used. A quick overview of metrics used and KFCV is given next.

### Sensitivity (Sens)

The metric that evaluates the ability of a model to predict true positives in each available class and is given in Eq. (2). It is also called as true positive rate (TPR).

2 $${\text{Sensitivity}} = {\text{TP}}/\left( {{\text{TP}} + {\text{ FN}}} \right)$$

### Specificity (Spec)

Specificity is a metric used to assess the ability of a model to predict true negatives in each available class and is given in Eq. (3). It is also called as false positive rate (FPR).

3 $${\text{Specificity}} = {\text{TN}}/\left( {{\text{T N}} + {\text{ F P}}} \right)$$

### Accuracy

Accuracy is defined as the no. of correct predictions divided by the total no. of input instances and is calculated using Eq. (4).

4 $${\text{Accuracy }} = \left( {{\text{TP }} + {\text{ TN}}} \right)/\left( {{\text{TP }} + {\text{ TN }} + {\text{ FP }} + {\text{ FN}}} \right)$$

### AUROC curve

The AUROC curve is an important evaluation statistic for binary classification tasks. The curve is a probability curve that plots TPR vs FPR at different thresholds, successfully distinguishing noise from signal. When compared to other values, the value at the upper (top) left side of the curve is deemed the best.

### Gini coefficient

The Gini coefficient represents a measure of the distribution of inequality in data. The value of Gini can be in-between 0 and 1: “1 indicating perfect data inequality and 0 perfect data equality”. It is given in Eq. (5).

5 $${\text{Gini}} = { 2}*{\text{ AUC}} - {1}$$

### Mathew’s correlation coefficient (MCC)

The MCC is a performance metric for quality elevation of a binary classification task. Its output is a value between − 1 and + 1, where + 1 indicates a perfect agreement between actual observation and prediction, − 1 represents total disagreement and 0 indicates no better than random prediction. The MCC is calculated using Eq. (6).

6 $$\mathrm{MCC}=\frac{\mathrm{TP}\cdot \mathrm{TN}-\mathrm{FP}\cdot \mathrm{FN}}{\surd (\mathrm{TP}+\mathrm{FP})(\mathrm{TP}+\mathrm{FN})(\mathrm{TN}+\mathrm{FP})(\mathrm{TN}+\mathrm{FN})}$$

### K-fold cross-validation

**Figure 6:**
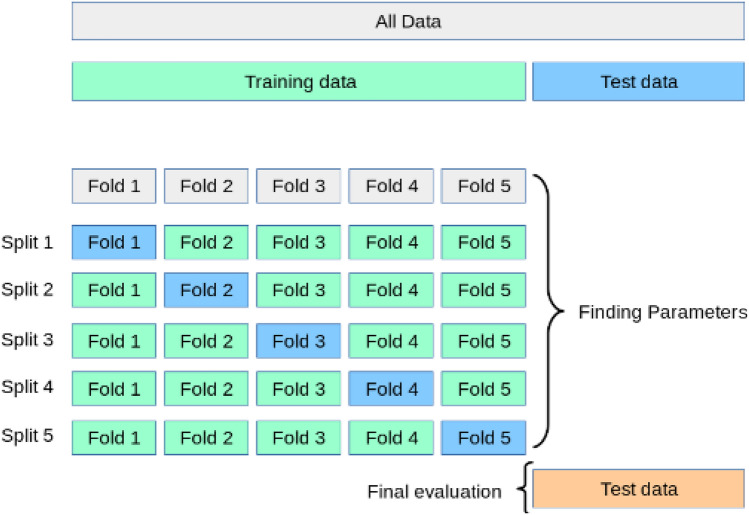
K-fold cross-validation technique.

A well-known technique known as KFCV is used to test model robustness and consistency^39^. The dataset is divided into k subsamples of equal size. For training the model, the k−1 subsamples are used in each iteration and the remaining one is used evaluating the model. As shown in Fig. 6, the process is carried out in such a way that each k sub-sample acts as a validation set just once^40^. Lastly, the sum of results from k-iterations is performed and an average is calculated as the mean accuracy of the model.

## Results

In this section, results of the proposed model are discussed in terms of evaluation parameters. In addition, an analysis of KFCV data is provided to determine the trustworthiness of the proposed ensemble model.

### Analysis of the accuracy and other performance metrics

The metrics as described in “Model evaluation” have been employed for evaluating the model performance. The results obtained for these metrics on the test data set are described in Table 5. The suggested model attained accuracy, AUC, sensitivity, specificity, Gini, and MCC of 0.9789, 0.984, 0.981, 0.987, 0.974, and 0.948, respectively, as shown in bold.

**Table 5:** Results in terms of accuracy and other performance metrics.

Model	Accuracy (%)	AUC	Gini	Sensitivity	Specificity	MCC
Decision tree	96.01	0.973	0.969	0.961	0.963	0.921
Neural network	93.98	0.943	0.938	0.956	0.951	0.918
SVM	95.78	0.962	0.946	0.972	0.966	0.896
adaBoost	96.32	0.978	0.939	0.969	0.982	0.901
RandomForest	96.21	0.971	0.976	0.949	0.949	0.927
Proposed model	97.89	0.984	0.981	0.987	0.974	0.948

**Figure 7:**
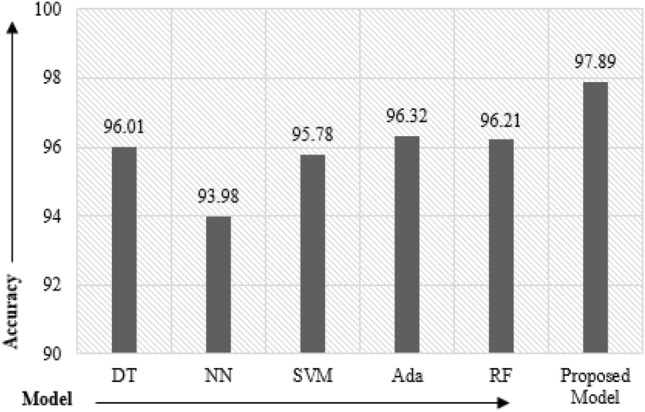
Performance comparison bar chart.

**Figure 8:**
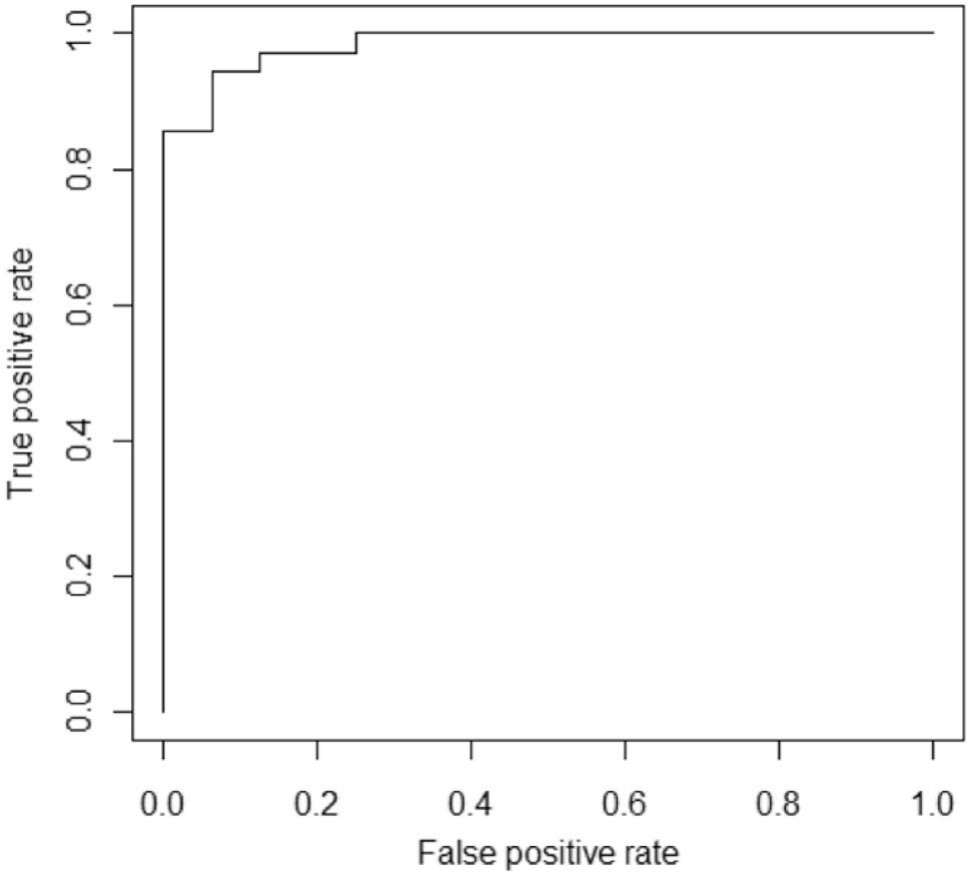
ROC curve of the proposed ensemble model.

Figure 7 depicts the accuracy plots of the proposed model and the base classifier in the form bar charts. The ROC curve is depicted in Fig. 8. As can be seen from Fig. 8, the proposed model achieved an AUROC value of 0.984. In general AUROC value of more than 0.9 is considered as outstanding and in the current study value obtained by is 0.984.The ROC curve clearly demonstrates that the proposed model consistently outperforms at all classification levels. The proposed model hence is skillful and outperforms the individual standard classifiers mentioned in Table 4.

### Cross-validation result analysis

**Figure 9:**
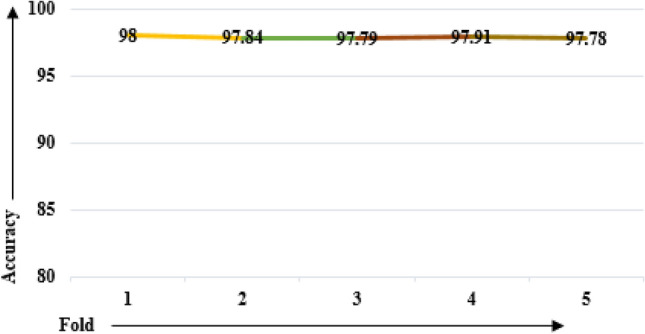
Accuracy line plot through K-fold cross validation.

Another factor to consider is the reliability of the proposed model, i.e., is the model free of overfitting and underfitting issues? Model overfitting indicates that the model performs well on training data but badly on testing data. On the other hand, underfitting demonstrates that the model performs badly on training and testing data sets. For analyzing the reliability of the proposed model, five-fold cross-validation was performed. The data set was splitted into five folds, four of which were used to train the model and one fold was kept for model evaluation. The accuracy obtained in each run is depicted in the form of a line plot as shown in Fig. 9. After running a fivefold CV, an average accuracy of 97.864% is recorded. The proposed voting-based ensemble model has performed consistently on all runs, as revealed by the fivefold CV findings.

## Conclusion

The ZIKV disease outbreak continues to this day^41^. Due to lack of vaccinations for its treatment and prevention, the disease continues to have a significant impact on people all over the world, particularly in third-world countries^42^. Given this, as well as the disease's recent outbreak in India, vaccine development for this disease is considered a high priority. Epitope-based peptide vaccines based on T-cell epitopes are already demonstrating promising results. Using a wet-lab experimental technique to detect TCEs is expensive and time-consuming^43,44^. In this study, an ML based ensemble computational model for the prediction of ZIKV TCEs has been proposed. The peptide sequences were obtained from the ViPR database. The physicochemical properties of amino acids were used to extract features, and the Burota algorithm was then used to choose significant features for model training. The proposed model was evaluated using a test set and achieved promising results. The proposed model obtained sensitivity, specificity, MCC, Gini, accuracy and AUC of 0.981, 0.987, 0.948, 0.974, 0.9789 and 0.984 respectively on test data set. The results are promising and indicate that the ensemble model proposed outperforms the existing standard ML classifiers, which include the RF, DT, SVM, NN, and AdaBoost. Furthermore, the proposed model's performance was found to be linear using the 5-FCV technique, with a mean accuracy of 0.97864. The epitopes predicted using the current model could serve as prospective peptide vaccine candidates for developing an epitope-based peptide vaccination against ZIKV. The model would save time for the scientific community working in vaccine development to screen active epitope candidates against inactive ones^45^. However it is pertinent to mention that the proposed model can only predict linear epitopes not the conformational ones. Nonetheless, there are several issues that can be addressed in future like exploring other physicochemical properties of amino acids and developing ensemble models based on various other cutting-edge ML classifiers to boost classification accuracy.

## Data Availability

Publicly available datasets were analyzed in this study. This data can be found here: http://www.viprbrc.org/ (accessed on 12th October 2021).
